# Time- and Computation-Efficient Calibration of MEMS 3D Accelerometers and Gyroscopes

**DOI:** 10.3390/s140814885

**Published:** 2014-08-13

**Authors:** Sara Stančin, Sašo Tomažič

**Affiliations:** Faculty of Electrical Engineering, University of Ljubljana, Ljubljana 1000, Slovenia; E-Mail: saso.tomazic@fe.uni-lj.si

**Keywords:** MEMS sensors, accelerometer, gyroscope, sensor calibration, general 3D sensor model, Simultaneous Orthogonal Rotation Angle (SORA)

## Abstract

We propose calibration methods for microelectromechanical system (MEMS) 3D accelerometers and gyroscopes that are efficient in terms of time and computational complexity. The calibration process for both sensors is simple, does not require additional expensive equipment, and can be performed in the field before or between motion measurements. The methods rely on a small number of defined calibration measurements that are used to obtain the values of 12 calibration parameters. This process enables the static compensation of sensor inaccuracies. The values detected by the 3D sensor are interpreted using a generalized 3D sensor model. The model assumes that the values detected by the sensor are equal to the projections of the measured value on the sensor sensitivity axes. Although this finding is trivial for 3D accelerometers, its validity for 3D gyroscopes is not immediately apparent; thus, this paper elaborates on this latter topic. For an example sensor device, calibration parameters were established using calibration measurements of approximately 1.5 min in duration for the 3D accelerometer and 2.5 min in duration for the 3D gyroscope. Correction of each detected 3D value using the established calibration parameters in further measurements requires only nine addition and nine multiplication operations.

## 1. Introduction

With the intensive development of different sensors based on microelectromechanical system (MEMS) technology [1–5], MEMS sensors are increasingly being used for simple and frequent measurements in various applications and fields, including the automotive industry [1–3], robotics [6], motion recognition and biomedicine [7–17], and virtual and/or augmented reality [18–21].

The MEMS sensors move together with the body to which they are attached, and thus, they directly reflect the motion of interest. The sensors are relatively small in size and lightweight; thus, they cause negligible physical interference with the observed motion. These sensors consume a small amount of energy, and hence, they offer a promising tool for tracking outdoor motion. Additionally, MEMS sensors are suitable for a wide range of commercial applications due to their low cost.

3D MEMS accelerometers measure acceleration along three orthogonal axes of sensitivity. The accelerometer is sensitive to the acceleration associated with gravity and changes in motion velocity. Although MEMS sensor technology is improving rapidly, such sensors do not yet provide for exact position determination because of different types of sensor inaccuracies (including the zero level offset, inaccurate sensitivity and misalignment of the sensor sensitivity axes), temperature drift and noise. If non-negligible acceleration is present in the motion, the typical obstacle is the correct deduction of the gravitational acceleration from the total measured acceleration. Because the position data are obtained by integrating the acceleration twice, even small errors in the determined direction of acceleration can cause the calculated position to deviate considerably from the true sensor position. Therefore, the widely available, low-cost, and somewhat inaccurate MEMS accelerometers are mainly suitable for monitoring the dynamics of the motion.

3D MEMS gyroscopes enable measurements of angular velocity in inertial space, and as such, they also provide for the tracking of angular orientation. Because the measured angular velocities represent simultaneous rotations, it is not appropriate to consider them sequentially. There are six possible sequences of three orthogonal rotations and thus six different angular orientations. Because rotations are generally not commutative, none of the six angular orientations provide the correct simultaneous rotation result. The rotation vector known as the Simultaneous Orthogonal Rotations Angle (SORA) is used to correctly interpret the values measured with the 3D gyroscope [22,23].

In this paper, we develop calibration methods for a 3D accelerometer and a 3D gyroscope. The aim of these calibration methods is to compensate for sensor inaccuracies including the zero level offset, inaccurate sensitivity and misalignment in the three sensitivity axes of the sensor. The calibration equations are derived considering a general noiseless model of a 3D sensor. We estimate the effect of the measurement noise on calibration separately, reducing the impact of the noise by averaging over a large number of calibration measurement samples.

### 1.1. Related Research

Various calibration methods for determining the values of the parameters that affect the accuracy of accelerometers and gyroscope sensors have been proposed to date for a variety of applications [24–36]. Methods for accelerometer calibration typically exploit the fact that the acceleration measured by a motionless sensor is equal to the gravitational acceleration [24–28]. The method proposed in [24] allows a variety of sensor inaccuracies to be considered, but it neglects the zero level offset. The method proposed in [25] is time consuming due to the 12 required sensor positions. The measurements collected in each position last for 10 min each, thus collectively requiring over 2 h of total calibration time. In methods proposed in [26–28], a number of measured values is used to build a cost function, which is subsequently minimized using various iterative procedures.

Methods for gyroscope calibration primarily include rotations of the sensor around different axes [29,30]. In [25], the authors consider Earth's rotation around its own axis for gyroscope calibration. Gyroscope calibration [25] is as time-consuming as accelerometer calibration [25].

Additional equipment has been suggested for the rotation and exact positioning of a sensor in different calibration positions [31–33]. The authors of [31,32] suggest the use of a robotic arm that moves the sensor to the required calibration position. The authors of [33] propose a specially designed device that allows for the controlled rotation of a sensor device containing 3D accelerometers and a 3D gyroscope around three orthogonal axes and thus allows for its exact positioning in different orientations. The methods for calibrating a 3D sensor proposed in [29,34] are based on the use of a reference optical system.

The method described in [30] allows for the calibration of a sensor device composed of a 3D accelerometer and 3D gyroscope and does not require any additional equipment. The zero level offset and sensitivity for each axis are determined based on measurements collected at rest in nine different positions and the application of Newton's method. Any misalignment in the accelerometer sensitivity axis is compensated for by rotating the sensor around two of the sensor sensitivity axes. The gyroscope zero level offsets are determined as the mean values measured at rest. The sensitivities and orientations of the axes are determined using controlled rotations of the sensor with a constant angular velocity around each sensitivity axis. The authors further suggest the use of Euler angles and a Kalman filter to determine the sensor orientation. A variety of methods have been proposed in which the sensor inaccuracies are treated as time-varying values [35,36] that are subsequently determined in an even more computationally demanding manner.

### 1.2. Problem Formulation and Research Contributions

MEMS sensors provide for the simple and frequent collection of a wide range of measurements due to their characteristic small size, light weight and low cost. Moreover, these measurements can be performed by an individual without any prior training. Hence, the time- and computation-consuming calibration procedures, as well as procedures whose efficiency is based on expensive additional equipment or reference systems, outweigh the reasonableness of the widespread use of MEMS sensors to a certain extent.

To avoid these limitations, we strive for simple and efficient methods (in terms of time and computational complexity) for calibrating a sensor device composed of MEMS sensors that do not require additional expensive equipment and that can be carried out by a casual user in a simple and brief manner.

The calibration methods proposed in this paper provide for the static compensation of measurement inaccuracies, which means that the sources of errors are treated as time invariant. The authors of [24] reported that dynamic accelerometer calibration procedures that consider sensor inaccuracies to vary with time do not significantly increase the accuracy of measurements. The dynamic methods also demand concurrent resources during sensor use and thus have a negative impact on the energy autonomy of the sensor device. Moreover, when the measured motion is highly dynamic, we can compensate for slowly changing time or temperature drift simply by implementing an adequate high pass filter.

In this paper, we assume that the response characteristics of a 3D accelerometer and 3D gyroscope are linear. Thus, the developed calibration methods are not appropriate for sensors with nonlinearities in which the measured values differ significantly from the values that are measured during calibration. Therefore, it should be determined prior to calibration whether the sensor response is linear. The methods developed here can only be used for a linear response. A nonlinear system response can be compensated for using several adaptive control strategies that have been reported in the literature (for example, the methods presented in [37,38]) could be used).

The calibration methods presented in this paper are sufficiently simple that they can easily be performed before each measurement, if necessary. It may be necessary to perform a calibration before making a measurement if the conditions of the measurement change in some way, such as if the operating temperature of the sensor changes. When the sensor is operating at a stable temperature, a calibration can be performed before making a measurement to compensate for sensor inaccuracies at that specific operating temperature. Compensating for sensor inaccuracies at a specific operating temperature prevents temperature deviations.

The primary contributions of this paper are as follows. We demonstrate that the values detected by both of the considered sensors (a 3D accelerometer and D gyroscope) can be treated using a general 3D sensor model. This model is based on assuming that the values detected by the sensor are equal to the projections of the measured values on the sensor sensitivity axes. Although this finding is trivial for 3D accelerometers, its validity for 3D gyroscopes is not immediately apparent. The angular velocities can be represented as vectors oriented in the direction of the rotational axis whose lengths are equal to the sizes of the angular velocities. However, these vectors cannot be simply treated as Euclidean vectors. In general, the sum of two angular velocity vectors in a 3D space does not determine their rotational sum. It is only by interpreting the detected rotations using the rotation vector SORA that we can apply the general model of a 3D sensor to a 3D gyroscope.

In contrast to some other calibration methods that have been developed [25–28], the methods presented in this paper rely on a small number of defined calibration measurements (six for a 3D accelerometer and four for a 3D gyroscope).

The six positions for calibrating a 3D accelerometer have already been presented (in [24], for example). In this paper, we demonstrate for the first time that arranging these six measurements into two triplets can be used to estimate all of the sensor inaccuracies considered with the general model of a 3D sensor. The estimated calibration parameters enable the complete static calibration of a 3D accelerometer.

The four positions for calibrating a 3D gyroscope have been previously presented (in [30], for example). However, while the authors of [30] claim that no additional equipment is necessary to calibrate the sensor, they also state that the sensor must be rotated at a constant angular velocity. This condition is impracticable without the use of additional equipment.

In this paper, we interpret the values detected with a 3D gyroscope using the rotation vector SORA, where the rotation vectors of the calibration rotations are obtained by averaging over the values of the measurement samples. This procedure enables us to consider non-constant angular velocities for calibration and actually perform the required calibration rotations manually.

### 1.3. Notions and Notations

The remainder of this paper is organized as follows. In Section 2, we present a generalized model of the 3D sensor common to 3D accelerometers and 3D gyroscopes. In the next two sections, we address each of the sensor's individual characteristics and propose methods for calibrating both types, *i.e.*, a 3D accelerometer in Section 3 and a 3D gyroscope in Section 4. The calibration results obtained for an example device are presented in Section 5. We conclude by emphasizing our findings and stating the benefits of the proposed calibration methods. In the subsequent sections, we obey the following notation rules: large bold letters denote matrices, small bold letters denote vectors, and large and small italics denote scalars.

## 2. 3D Sensor

A 3D sensor is a device that measures a given physical quantity in a 3D space. To achieve this objective, the sensor is composed of three mutually perpendicular sensitivity axes, which we denote as *x*, *y* and *z*. These axes form the intrinsic coordinate system (*x y z*) of the sensor.

In this work, we present a generalized noiseless model of a 3D sensor that provides a link between the measured physical quantity and the values detected by the sensor. This generalized model is common to the specific sensor models (the 3D accelerometer and 3D gyroscope) discussed in subsequent sections.

### 2.1. Noiseless 3D Sensor Model

Let **q** be the column vector of the measured physical quantity in the intrinsic coordinate system of the sensor:

(1) $$\text{q}=\left[\begin{array}{c} q_{x}\\ q_{y}\\ q_{z} \end{array}\right]$$

and let **q_s_** be a column vector of the values detected by a 3D sensor along its three sensitivity axes:

(2) $${\text{q}}_{\text{s}}=\left[\begin{array}{c} q_{s,x}\\ q_{s,y}\\ q_{s,z} \end{array}\right]$$

In the noiseless model, the accuracy of the measured values Equation (2) depends on the zero level offset, sensitivity, and alignment of the three sensitivity axes of the sensor.

The zero level offset is the value detected by the sensor when the measured physical quantity is equal to zero. We denote the zero level offsets of the three sensitivity axes as *q_o,x_*, *q_o,y_* and *q_o,z_*. The sensitivity of the sensor is equal to the ratio between the change in the detected value and the change in the real value, assuming that the sensor characteristic is full-scale linear. We denote the sensitivities of the three axes as *k_x_*, *k_y_* and *k_z_*.

Due to imprecise manufacturing, the sensor sensitivity axes can be misaligned and thus deviate from the sensor coordinate axes. We consider the orientation of each sensor sensitivity axis to be independent of the remaining two axes. Let **v_x_**, **v_y_** and **v_z_** denote the directional unit vectors that represent the orientation of the sensitivity axes. We also assume that values detected by a 3D sensor represent the projections of the measured physical quantity on each of the three sensitivity axes of the device. The value detected along each axis is thus equal to the sum of the zero level offset and the projection of the real value on that axis multiplied by the axis sensitivity. We write:

(3) $$\begin{array}{c} q_{s,x}=k_{x}\left({\text{v}}_{\text{x}}\cdot \text{q}\right)+q_{o,x}\\ q_{s,y}=k_{y}\left({\text{v}}_{\text{y}}\cdot \text{q}\right)+q_{o,y}\\ q_{s,z}=k_{z}\left({\text{v}}_{\text{z}}\cdot \text{q}\right)+q_{o,z} \end{array}$$

The above three equations can be combined into the following single expression:

(4) $${\text{q}}_{\text{s}}=\text{K}\cdot \text{V}\cdot \text{q}+{\text{q}}_{\text{o}}$$

where **K** is a 3 × 3 matrix of sensor sensitivities:

(5) $$\text{K}=\left[\begin{array}{ccc} k_{x} & 0 & 0\\ 0 & k_{y} & 0\\ 0 & 0 & k_{z} \end{array}\right]$$

**V** is a 3 × 3 matrix in which the rows are equal to the three directional vectors **v_x_**, **v_y_** and **v_z_**, and **q_o_** is a column vector of zero level offsets:

(6) $${\text{q}}_{\text{o}}=\left[\begin{array}{c} q_{o,x}\\ q_{o,y}\\ q_{o,z} \end{array}\right]$$

We obtain the expression for calculating the measured physical quantity in the coordinate system of the sensor from the values detected by a 3D sensor by rearranging Equation (4) as:

(7) $$\text{q}={\text{C}}_{\text{s}}\cdot ({\text{q}}_{\text{s}}-{\text{q}}_{\text{o}})$$

where **C_s_** is a 3 × 3 calibration matrix containing the nine parameters required for sensor calibration and is given by the following expression:

(8) $${\text{C}}_{\text{s}}={\left(\text{K}\cdot \text{V}\right)}^{-1}$$

To calibrate a 3D sensor defined according to the general 3D sensor model Equation (7), we must determine the values of 12 parameters, *i.e.*, the nine elements of the calibration matrix **C_s_** and the three elements of the zero level offset vector **q_o_**. To achieve this goal, we must obtain 12 equations from measurements of known reference physical quantities.

The values of the three sensor sensitivities given in **K** and the nine orientation values in **V** are not required for calibration. However, if necessary, these values can be obtained from the calibration matrix by considering the norms of the directional unit vectors:

(9) $$\left\|{\text{v}}_{\text{x}}\right\|=1$$

(10) $$\left\|{\text{v}}_{\text{y}}\right\|=1$$

(11) $$\left\|{\text{v}}_{\text{z}}\right\|=1$$

Because Equations (9)–(11) represent three of the required 12 equations, the remaining nine are provided by Equation (8).

### 2.2. Computational Complexity of Calculating the Correct Value

The calibration parameters given by **q_o_** and **C_s_** are used to correct the value detected by the 3D sensor. The correct value of the measured quantity **q** is obtained by considering Equation (7). Given **C_s_** and **q_o_**, correction of each detected value **q_s_** according to Equation (7) requires nine addition operations and nine multiplication operations. Thus, data capture with an example capture frequency of 1000 samples per second requires 9000 addition operations and 9000 multiplication operations per second.

## 3. 3D Accelerometer

3D accelerometers measure accelerations along three mutually perpendicular sensitivity axes. Because they are sensitive to gravitational acceleration and proper accelerated motion, accelerometers have several different applications in many areas [1,2,6–10,12,16–21]. Together with gyroscopes, accelerometers form inertial navigation systems used for determining the position, velocity, and orientation of an object in a reference space [39–44]. Highly accurate inertial navigation systems are used to track motion and provide navigation for missiles, aircraft, ground and underwater vehicles, and robots [42–44].

The values detected by a 3D accelerometer represent projections of the measured acceleration on each of the three sensitivity axes of the device. Due to their sensitivity to gravitational acceleration, when at rest on an even horizontal surface, the sensor exhibits an acceleration of 1 g along the sensitivity axis pointing upward in the direction normal to the horizontal surface. This feature makes it easy to determine the orientation of the sensor with respect to the direction of gravitational acceleration vector in the coordinate system of the accelerometer. The accelerometer must be at rest or moving with a negligible acceleration relative to the size of the gravitational acceleration when determining the orientation in such a way.

Although MEMS sensor technology is improving rapidly, 3D MEMS accelerometers do not provide for exact position determination [40,44]. When non-negligible acceleration is present in the motion, the gravitational acceleration is often not correctly subtracted from the total measured acceleration because of various sensor inaccuracies, temperature drift and noise. Such an improperly subtracted gravitational acceleration is reflected in a deviation of the determined acceleration direction from the real direction of the accelerated motion. Because the position data are obtained from integrating the acceleration twice, even small errors in the determined acceleration direction can result in significant deviations of the calculated position from the true sensor position.

Thus, the widely available, low-cost, and somewhat inaccurate MEMS accelerometers are most suitable for monitoring motion dynamics. Instead of determining the absolute motion values to investigate motion dynamics, the attention is dedicated to the perception of motion changes, aiming to create an efficient framework for data analysis. Motion dynamics analysis has been shown to be an efficient strategy for motion recognition and evaluation [11,13–15,17].

Regardless of the planned application of the MEMS accelerometer, the accuracy of the captured data is essential for relevant and comparable results and to enable an efficient and comprehensive analysis. Depending on the sensor manufacturing process, different sources of inaccuracies can cause measurement errors. The proposed 3D accelerometer calibration procedure is designed to compensate for the zero level offsets, inaccurate sensitivities, and misalignments of the sensor sensitivity axes in a time- and computation-efficient manner.

In the following sections, we consider an accelerometer that measures acceleration in units of gravitational acceleration, g. Furthermore, we consider all of the measured and detected values to be normalized by 1 g.

### 3.1. Specific Sensor Model

In applying the general model of a 3D sensor presented in the previous section, we create a specific model of a 3D accelerometer. We use **a** to denote the vector of the measured acceleration in the sensor's intrinsic coordinate system (*x y z*). From Equation (7), **a** is determined from the values detected by the accelerometer in accordance with the following expression:

(12) $$\text{a}={\text{C}}_{\text{s}}\cdot ({\text{a}}_{\text{s}}-{\text{a}}_{\text{o}})$$

where **a_s_** denotes the vector of acceleration detected by the 3D sensor along its three sensitivity axes, **C_s_** is the accelerometer calibration matrix Equation (8), and **a_o_** is the vector of the zero level offsets of the three sensitivity axes Equation (6).

### 3.2. Calibration Procedure

The accelerometer calibration exploits the fact that the size of the measured acceleration is constant and equal to the gravitational acceleration for the sensor at rest. Thus, we can estimate the values of the nine elements of the calibration matrix **C_s_** and the three elements of the zero level offset vector **a_o_** in Equation (12) using the known values of the acceleration vector and the values detected by the sensor in a number of different sensor orientations.

**Figure 1.**
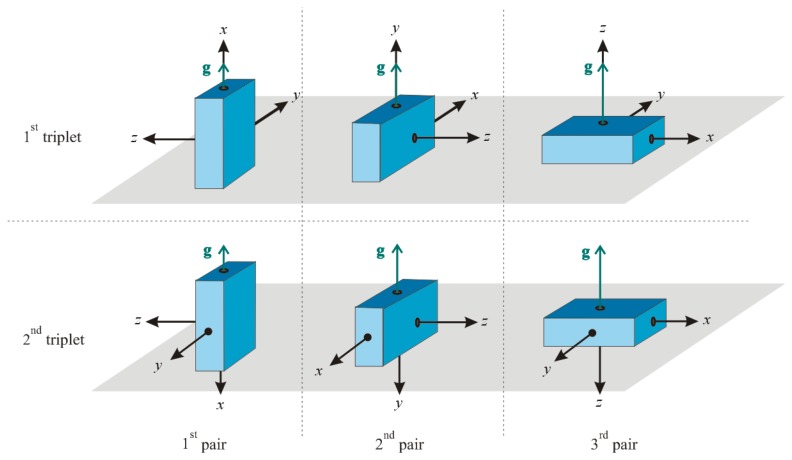
Orientations of the 3D accelerometer during the six calibration measurements. For the first measurement triplet, each of the three sensor coordinate axes *x*, *y* and *z* is, in turn, aligned with the direction of **g**. For the second measurement triplet, each of the three sensor coordinate axes *x*, *y* and *z* is, in turn, aligned with the direction opposite to the direction of **g**. The first measurements in both triplets together represent the calibration measurement pair for the *x*-axis; the same holds for the remaining two axes.

To estimate the values of the calibration parameters contained in **C_s_** and **a_o_**, we perform measurements while the sensor is at rest on an even horizontal surface in six different orientations. We divide these six measurements into two triplets, as illustrated in Figure 1. The illustrated device contains the sensor, and the coordinate axes of the device are aligned with the sensor coordinate axes. If the device containing the sensor has unevenly rounded surfaces, a specially designed casing for the device must be used to accurately place the sensor at the required positions.

For the first three measurements, each of the three sensor coordinate axes *x*, *y* and *z* is, in turn, aligned with the direction of **g**. We denote the 3 × 3 gravitational matrix as **A**_+_. The columns of matrix **A**_+_ are the gravitational acceleration vectors in the sensor's intrinsic coordinate system for each of the three measurements, and the rows represent the intrinsic coordinate axes. We write:

(13) $${\text{A}}_{\text{+}}=\left[\begin{array}{ccc} 1 & 0 & 0\\ 0 & 1 & 0\\ 0 & 0 & 1 \end{array}\right]=\text{I}$$

We further denote a 3 × 3 matrix of the corresponding values detected by the sensor for each of the three calibration measurements as **A_s_**_+_. The columns of matrix **A_s_**_+_ are equal to the vectors of the detected acceleration values for each of the three measurements, and the rows represent the sensitivity axes.

Similarly, for the second measurement triplet, each of the three sensor coordinate axes *x*, *y* and *z* is, in turn, aligned with the direction opposite to the direction of **g**. In this manner, we combine the gravitational acceleration vectors in a 3 × 3 matrix **A**_−_:

(14) $${\text{A}}_{-}=\left[\begin{array}{ccc} -1 & 0 & 0\\ 0 & -1 & 0\\ 0 & 0 & -1 \end{array}\right]=-\text{I}$$

and the values detected by the sensor in a 3 × 3 matrix **A_s_**_−_.

According to the specific model of the 3D accelerometer Equation (12), we now write:

(15) $$\text{I}={\text{C}}_{\text{s}}\cdot ({\text{A}}_{\text{s}\;\text{+}}-{\text{A}}_{\text{o}})$$

(16) $$-\text{I}={\text{C}}_{\text{s}}\cdot ({\text{A}}_{\text{s}\;-}-{\text{A}}_{\text{o}})$$

where **A_o_** denotes a 3 × 3 matrix in which all columns are equal to the zero level offset vector **a_o_**.

Subtracting Equation (16) from Equation (15) yields:

(17) $$2\;\text{I}={\text{C}}_{\text{s}}\cdot ({\text{A}}_{\text{s}\;\text{+}}-{\text{A}}_{\text{s}\;-})$$

and after rearrangement:

(18) $${\text{C}}_{\text{s}}=2\;{\left({\text{A}}_{\text{s}\;\text{+}}-{\text{A}}_{\text{s}\;-}\right)}^{-1}$$

The elements of calibration matrix **C_s_** represent nine of the required twelve calibration parameters. We sum Equations (15) and (16) to determine the remaining three parameters (elements of vector **a_o_**). After rearrangement, we obtain:

(19) $${\text{A}}_{\text{o}}=\frac{{\text{A}}_{\text{s}\;\text{+}}+{\text{A}}_{\text{s}-}}{2}$$

Each column of matrix **A_o_** represents the zero level offset vector **a_o_** obtained with one pair of measurements. In each measurement pair, one of the sensor axes is aligned along the direction of and direction opposite to the direction of **g**.

Ideally, for a noiseless sensor with a constant zero offset, all three columns will be equal, and any of these columns could be used to determine **a_o_**.

However, the columns of **A_o_** may differ when considering noisy measurements. Instead of determining vector **a_o_** by considering only one pair of measurements (one column of **A_o_**), we obtain more accurate results by averaging the values obtained for all three pairs as:

(20) $${\text{a}}_{\text{o}}=\frac{{\text{A}}_{\text{o}}\cdot \text{i}}{3}=\frac{\left({\text{A}}_{\text{s}\;\text{+}}+{\text{A}}_{\text{s}\;-}\right)\cdot \text{i}}{6}$$

where **i** is a vector of ones:

(21) $$\text{i}=\left[\begin{array}{c} 1\\ 1\\ 1 \end{array}\right]$$

To explain the physical meaning of Equations (18) and (20), let us consider two measurements for one of the sensor coordinate axes, the *x*-axis, for example. These two measurements are the first measurements for both the measurement triplets. We can draw the following conclusion about the projection of **g** onto the *x*-axis. In one measurement, the zero level offset is added to the measured value, whereas the zero level offset is subtracted in the other measurement. The sensitivity and alignment of the sensitivity axis have equal effects on both measurements. The projection of **g** onto the *x*-axis changes sign between the two measurements.

The same consideration also holds for the other two axes, *y* and *z*. Thus, it follows that by subtracting the measured values for both measurement triplets Equation (18), we obtain the sensitivities and alignments, whereas adding these values Equation (20) yields the zero level offset.

Using Equations (18) and (20), we can estimate the values of all 12 calibration parameters and subsequently use these values in real time during other measurements to compensate for inaccuracies, *i.e.*, to calculate the true **a** from the detected **a_s_** values using Equation (12).

In the presented model of the 3D accelerometer, Equation (12), we neglected the influence of noise. The noise that is present in practice introduces an error in the estimated values of the 12 calibration parameters. However, during each calibration measurement, the device remains at rest for a sufficiently long period of time, thus enabling multiple sample captures. In this manner, we can obtain the columns of matrices **A_s_**_+_ and **A_s_**_−_ by averaging a large (≫1) number of samples captured for the associated calibration measurement.

In averaging multiple samples, the power of the noise is reduced by a factor of the number of the captured samples. Depending on the number of captured samples, the noise that affects the calibration could be substantially lower than the noise that affects each individual measurement. In Appendix A, we present an analytical derivation of the ratio of the power of the measurement noise to the variance of the error from the noisy calibration *NER* for a 3D accelerometer. The solution presented shows that *NER* depends on the number of samples collected and the magnitude of the measured acceleration. We can ensure that an adequate number of samples are collected by requiring that *NER* is sufficiently large, for example, larger than 6 dB, for all of the values in the measurement range of the sensor.

The total number of samples collected, together with the sampling frequency, determines the total calibration time. A large number of samples can be obtained in a short period of time if the sensor supports a high sample frequency.

## 4. 3D Gyroscope

3D gyroscopes offer measurements of angular velocity in inertial space around three mutually perpendicular sensitivity axes. Similar to accelerometers, gyroscopes have several different applications in many fields [1–3,7,10,11,13–16,39–44].

By providing for angular velocity measurements, a 3D gyroscope also allows for the orientation of an object to be determined. In general, the orientation of a rigid body is the position of its coordinate system observed relative to a reference coordinate system with the same origin. The orientation can be described by a rotation that would move the rigid body's coordinate system, which is initially aligned with the reference coordinate system, to its new position. When working with gyroscope measurements, we consider the gyroscope as the rigid body and the inertial space coordinate system as the reference system. The measured angular velocity determines the rotation required to move the sensor to its new position.

Rotations are typically not commutative. The three simultaneous angular velocities detected around each sensitivity axis of a 3D gyroscope cannot be considered as sequential rotations. There exist six different sequences of three rotations around different axes. Each of these six sequences determines a different orientation, none of which correspond to the orientation that it is obtained by considering the three rotations as simultaneous.

Angular velocities can be represented as vectors oriented in the direction of the rotational axis with a length equal to the size of the angular velocity. However, these vectors cannot be unreservedly treated in the same manner as Euclidian vectors. In general, the sum of two angular velocity vectors in a 3D space does not determine their rotational sum.

Due to the exposed specificities of a 3D gyroscope sensor, it is not self-evident that the angular velocity values detected by this sensor represent projections of the measured angular velocity on each of the three sensitivity axes of the device. Thus, the measurements of a 3D gyroscope must be correctly interpreted before defining its proper specific model.

In the following sections, we consider a gyroscope that measures angular velocity in units of °/s. Furthermore, we consider all of the measured and detected values to be normalized by 1°/s.

### 4.1. Interpretation of Gyroscope Measurements

Let **ω** be the angular velocity vector, *i.e.*, a vector oriented in the direction of the axis of rotation with a length equal to the angular velocity around that axis. In [22,23] we have already demonstrated that the coordinates *ω_x_*, *ω_y_* and *ω_z_* of this vector in the 3D gyroscope's intrinsic coordinate system:

(22) $$\text{ω}=\left[\begin{array}{c} \omega_{x}\\ \omega_{y}\\ \omega_{z} \end{array}\right]$$

are equal to the angular velocities measured using the 3D gyroscope when its sensitivity axes are aligned with its intrinsic coordinate system axes. Because the coordinates *ω_x_*, *ω_y_* and *ω_z_* are the projections of the angular velocity vector **ω** onto the coordinate axes of the 3D gyroscope as well as the values detected by the gyroscope along these axes, we can conclude that a 3D gyroscope detects the projection of the measured angular velocity on the sensor sensitivity axes.

### 4.2. Specific Sensor Model

As shown in the previous section, the assumption that a 3D sensor detects the projections of the measured quantity onto its sensitivity axis (see Section 2.1) is valid for a 3D gyroscope. Thus, the generalized model of a noiseless 3D sensor Equation (7) is also applicable to a 3D gyroscope. Considering Equation (7) and using **ω** and **ω_s_** for the column vectors of the actual and detected angular velocities, respectively, in the sensor coordinate system (*x y z*), we write:

(23) $$\text{ω}={\text{C}}_{\text{s}}\cdot ({\text{ω}}_{\text{s}}-{\text{ω}}_{\text{o}})$$

In the above expression, **ω_o_** is the vector of the zero level offsets of the sensitivity axes and **C_s_** is the calibration matrix, which, in accordance with Equation (8), is dependent on the effects of the inaccurate sensitivities and orientations of the sensor sensitivity axes.

### 4.3. Calibration Procedure

The following calibration procedure is designed to compensate for the effects of the zero level offset, inaccurate sensitivity, and orientation of the 3D gyroscope sensitivity axes. Similarly to the calibration of the 3D accelerometer, the main requirements are time and computational efficiency.

**Figure 2.**
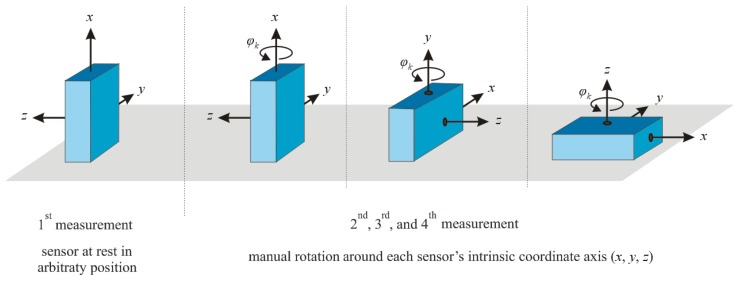
Orientations of the 3D gyroscope during the four calibration measurements. The first calibration measurement (**left**) is performed with a sensor at rest in an arbitrary position. During each of the remaining three measurements (**right**), the sensor is manually rotated on an even horizontal surface around a different coordinate system axis (*x*, *y* or *z*).

To calibrate a 3D sensor, we must estimate the values of 12 calibration parameters, *i.e.*, the values of the nine elements of calibration matrix **C_s_** and the three elements of the zero level offset vector **ω_o_** in Equation (23). We perform four measurements to achieve this objective for the 3D gyroscope. The orientations of the sensor during these measurements are illustrated in Figure 2. The illustrated device contains the sensor, and the coordinate axes of the device are aligned with the sensor coordinate axes.

The vector **ω_o_** of the zero level offsets of the sensitivity axes for the 3D gyroscope can be easily obtained by performing a single measurement when the sensor is at rest in an arbitrary position. This approach is commonly used to estimate the zero level offset vector **ω_o_** because for a noiseless sensor, the values detected by the sensor along each axis when the sensor is at rest are equal to each of the zero level offsets for these axes. Thus, for the first calibration measurement, we write:

(24) $${\text{ω}}_{\text{o}}={\text{ω}}_{\text{s}}$$

The remaining three measurements are performed to estimate the values of the nine elements of the calibration matrix **C_s_** During each of these measurements, the sensor is manually rotated on an even horizontal surface, each time around a different coordinate axis (*x*, *y* or *z*). We combine the angular velocities of the sensor in its intrinsic coordinate system during each of these three calibration measurements into a 3 × 3 matrix denoted by **Ω**. The columns of matrix **Ω** are equal to the vectors of the sensor angular velocity around coordinate axes *x*; *y* and *z*; and the rows represent the coordinate axes in the stated order:

(25) $$\text{Ω}=\left[\begin{array}{ccc} {\text{ω}}_{\text{k},\;\text{x}} & {\text{ω}}_{\text{k},\;\text{y}} & {\text{ω}}_{\text{k},\;\text{z}} \end{array}\right]=\left[\begin{array}{ccc} \omega_{k,\;x} & 0 & 0\\ 0 & \omega_{k,\;y} & 0\\ 0 & 0 & \omega_{k,\;z} \end{array}\right]$$

Because the sensor rotations are performed manually; the angular velocity is not constant during each of the three calibration measurements. However; we assume that the rotational axis does not change for each of these measurements. In such a case; we can interpret the measured values using the SORA vector [22,23]. Therefore; the addition of the angular velocity vectors does indeed represent the rotational sum of the added vectors. Thus; the elements of matrix **Ω** Equation (25) represent the average values of the respective angular velocities in the calibration measurement. These elements can be determined by considering the total time and rotation angle for each of the three measurements.

We further combine the values detected by the 3D gyroscope during each of these three measurements into a 3 × 3 matrix **Ω_s_**. The columns of matrix **Ω_s_** are equal to the vectors of the detected angular velocity, and the rows represent the sensitivity axes. Because the axis of rotation during each individual calibration measurement does not change, we can obtain the vector of detected values for each measurement by simply averaging all of the vectors of values detected for the corresponding measurement.

In accordance with Equation (23), we write

(26) $$\text{Ω}={\text{C}}_{\text{s}}\cdot ({\text{Ω}}_{\text{s}}-{\text{Ω}}_{\text{o}})$$

where **Ω_o_** denotes a 3 × 3 matrix in which all columns are equal to **ω_o_**. From Equation (26), we can obtain the expression for calculating the calibration matrix **C_s_** as

(27) $${\text{C}}_{\text{s}}=\text{Ω}\cdot {\left({\text{Ω}}_{\text{s}}-{\text{Ω}}_{\text{o}}\right)}^{-1}$$

Using Equations (24) and (27), we can estimate the values of all 12 calibration parameters required for the static compensation of the inaccuracies of the 3D gyroscope considered in the general model of the 3D sensor.

In the presented model of the 3D gyroscope, we neglected the influence of noise. When working with noisy measurements, the number of desired samples can be obtained by measuring the device at rest (for the first calibration measurement) for a sufficiently long period of time. For the remaining three measurements, when we rotate the sensor manually, a greater number of samples for a nearly equal angular velocity require a larger rotation angle, which means that the calibration results will be more accurate for larger calibration angles.

We ensure that an adequate number of samples is collected by considering the relationship between the error from the calibration and error from the noise. In Appendix B, we present an analytical derivation of the ratio of the power of the measurement noise to the variance of the error from the noisy calibration *NER* for a 3D gyroscope. The solution presented shows that *NER* depends on the number of samples collected, the measured values and the angular velocities in the calibration. We ensure that an adequate number of samples is collected by requiring that *NER* is sufficiently large, for example, larger than 6 dB, for all of the values in the measurement range of the sensor.

The total number of samples collected, together with the sampling frequency, determines the total calibration time.

## 5. Calibration Measurements and Results

### 5.1. Sensor Device

**Figure 3.**
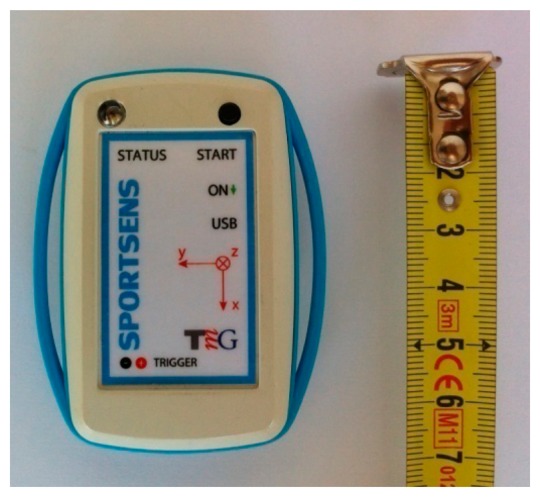
Wireless sensor device used to test the proposed calibration procedure. The device includes a 3D accelerometer and 3D gyroscope and thus enables the capturing of acceleration and angular velocity data along the three coordinate system axes (*x*, *y* and *z*).

To test the proposed calibration procedure, we used a wireless sensor device that combines a 3D accelerometer and 3D gyroscope to enable the capturing of acceleration and angular velocity data along the three coordinate axes of the device coinciding with the three coordinate axes of both sensors. This device also includes a 3.77 GB memory card, microcontroller, and power battery supply. The device measures 69 × 54 × 15 mm in size and has a weight of 30 g. Figure 3 displays the device.

The integrated MEMS 3D accelerometer LIS331HH is manufactured by STMicroelectronics (Geneva, Switzerland) [45] and is implemented in the form of an integrated 3 × 3 × 1 mm circuit with 16 pins. The integrated MEMS 3D gyroscope ITG3200-3 is manufactured by InvenSense (Sunnyvale, CA, USA) [46] and is implemented in the form of an integrated 4 × 4 × 0.9 mm circuit with 24 pins. This device consists of three independent MEMS gyroscopes positioned in a mutually orthogonal manner. Both sensors are positioned such that their coordinate system axes coincide with the coordinate axes of the device illustrated in Figure 3.

The integrated accelerometer provides output in units of gravitational acceleration g = 9.81 m/s^2^. For all performed measurements, the measurement range of the accelerometer was set to ±24 g, and the gyroscope measured in a ±2000°/s range. Both sensors provide 16-bit outputs. The sampling rates of both sensors analog-to-digital converters were set to *f_s_* = 1000 Hz. The sample frequency *f_s_* was set as high as possible to allow multiple samples to be obtained in a shorter period of time and thus to enable time-efficient calibration. The cut-off frequencies of the digital low-pass filter for the accelerometer and gyroscope were set to *f_c_* = 292 Hz and *f_c_* = 188 Hz, respectively.

**Figure 4.**
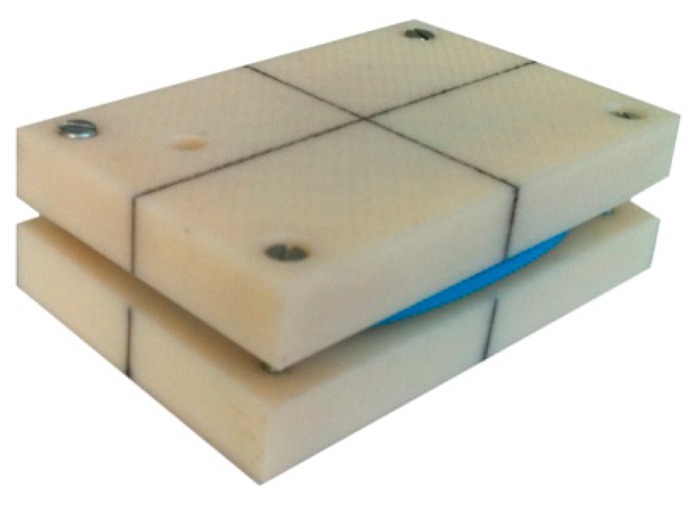
Casing for the sensor device calibration. The casing enables all required calibration positions and rotations of the sensor device.

Due to an unevenly rounded surface on the sensor device (see Figure 3), the device itself could not be positioned to remain still in all of the orientations required for accelerometer calibration or be reliably rotated around a single coordinate axes, as is necessary for gyroscope calibration. To overcome this obstacle, we used a dedicated and specially designed low-cost casing produced using a 3D printer. The inner cavity of the casing was designed to fit the device, and the square shape and outer edges of the casing parallel to the coordinate axes of the calibrated device made the required calibration measurements possible. Figure 4 displays the casing for the sensor device calibration.

The specially designed casing can be used to ensure that the position of the device remains fixed in any orientation in which one of the device axes is perpendicular to the horizontal surface on which the device is placed. This procedure is required for all of the six 3D accelerometer calibration positions and for the first position in calibrating a 3D gyroscope. The casing can also be used to accurately manually rotate the sensor around its intrinsic coordinate axes. This procedure is required for the remaining three measurements in calibrating a 3D gyroscope. To accurately rotate the sensor around its coordinate axis that is perpendicular to the even horizontal surface on which the sensor is placed, one of the vertical faces of the sensor is initially pressed along an auxiliary surface (such as a wall). The sensor is then rotated on the horizontal surface on which it is placed. Finally, one of the vertical faces of the sensor is again pressed along the auxiliary surface. For this procedure, the calibration rotation angle can be equal to a multiple of 90°. In the absence of an auxiliary surface, the initial position can be marked and used to place the sensor in its final position after rotation.

### 5.2. 3D Accelerometer Calibration

The 3D accelerometer was calibrated in accordance with the procedure presented in Section 3.

#### 5.2.1. Measurements

**Figure 5.**
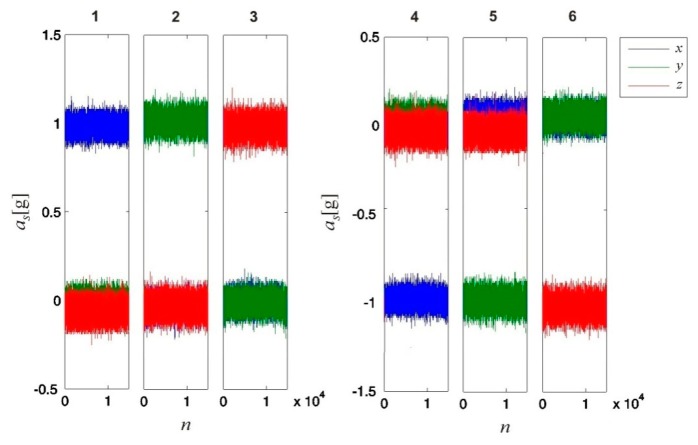
Values detected by the 3D accelerometer for all six calibration measurements. During each measurement, the sensor was positioned on an even horizontal surface in a different orientation. The sensor orientations were set such that the sensor's intrinsic coordinate axes (*x*, *y* and *z*) were aligned in turn along (measurements 1, 2, and 3) and opposite to (measurements 4, 5, and 6) the direction of gravitational acceleration **g**. The data were captured at a sampling rate *f_s_* = 1000 Hz.

During each of the six calibration measurements, the sensor was alternately positioned such that its intrinsic axes (*x*, *y* and *z*) were aligned along and opposite to the direction of **g**. Each of these six measurements required 15 s, resulting in a total duration of 1.5 min. For each measurement (sampled at *f_s_* = 1000 Hz), we obtained *N_a_* = 15,000 data samples. Figure 5 presents the values detected by the sensor.

The calibration time is determined by considering the effect of noise on the error in the estimated calibration parameters. The analytic solution presented in Appendix A shows that for the considered 3D accelerometer (±24 g range), for any value *N_a_* > 4000, the ratio between the power of the measurement noise to the variance of the error from the noisy calibration *NER* is larger than 6 dB. For convenience, we rounded the measurement time to a quarter of a second.

The 3D accelerometer investigated produces 16-bit outputs within the ±24 g range. Each LSB thus accounts for 0.00073 g. The variance of the quantization noise is (0.00073 g)^2^/12 = 45 ng^2^. The estimated variance of the sensor noise from the measurements is 0.0025 g^2^. The total variation of the noise is equal to the sum of both variances, showing that the variance of the quantization noise is negligible in this case.

For *N_a_* measurement samples, the variance of the noise is reduced by the factor *N_a_*. For *N_a_* = 15,000 samples, the total variance of the noise is hence 0.0025/15,000 = 0.17 ug^2^. Thus, the total standard deviation of the noise is 0.00041 g, from which we can conclude that the measurements are accurate up to three decimal places. These values are meaningful up to four decimal places. We present the 3D accelerometer calibration results at this precision.

#### 5.2.2. Results

The average detected accelerations **A_s_**_+_ and **A_s_**_−_ were:

(28) $${\text{A}}_{\text{s}+}=\left[\begin{array}{ccc} 0.9835 & -0.0317 & 0.0041\\ -0.0209 & 1.0201 & -0.0030\\ -0.0614 & -0.0263 & 0.9897 \end{array}\right]$$

(29) $${\text{A}}_{\text{s}-}=\left[\begin{array}{ccc} -1.0148 & 0.0158 & -0.0007\\ 0.0019 & -1.0279 & 0.0133\\ -0.0582 & -0.0718 & -1.0625 \end{array}\right]$$

Inserting the values of **A_s_**_+_ Equation (28) and **A_s_**_−_ Equation (29) into Equations (18)–(20) yields the following values for the calibration parameters:

(30) $${\text{C}}_{\text{s}}=\left[\begin{array}{ccc} 1.0011 & 0.0233 & -0.0022\\ 0.0093 & 0.9766 & 0.0078\\ 0.0014 & -0.0216 & 0.9744 \end{array}\right]$$

(31) $${\text{A}}_{\text{o}}=\left[\begin{array}{ccc} -0.0156 & -0.0077 & 0.0013\\ -0.0120 & -0.0042 & 0.0049\\ -0.0595 & -0.0488 & -0.0363 \end{array}\right]$$

(32) $${\text{a}}_{\text{o}}=\left[\begin{array}{c} -0.0073\\ -0.0034\\ -0.0484 \end{array}\right]$$

The matrices of the 3D accelerometer sensitivities **K** and orientation **V** obtained using the values of **C_s_** Equation (30) and considering the norms of the directional vectors Equations (9)–(11) are:

(33) $$\text{K}=\left[\begin{array}{ccc} 0.9994 & 0 & 0\\ 0 & 1.0241 & 0\\ 0 & 0 & 1.0263 \end{array}\right]$$

(34) $$\text{V}=\left[\begin{array}{ccc} 0.9997 & -0.0238 & 0.0024\\ -0.0093 & 0.9999 & 0.0080\\ -0.0016 & 0.0222 & 0.9998 \end{array}\right]$$

The matrix of the angles between the sensor coordinate and sensitivity axes **Ψ** that follow from **V** Equation (34) is:

(35) $$\text{Ψ}=\mathrm{acos}(\text{V})=\left[\begin{array}{ccc} 1.3702 & 91.3633 & 89.8626\\ 90.5301 & 0.7003 & 90.4577\\ 90.0909 & 88.7306 & 1.2726 \end{array}\right]$$

In the above expression, all angles are given in degrees. The rows of matrix **Ψ** represent the sensor sensitivity axes, and its columns represent the sensor coordinate axes. Equations (32), (33), and (35) indicate that the largest inaccuracy in the detected value considering the zero level offset and sensitivity can be attributed to the inaccuracies of the sensor *z*-axis. However, both of these values are relatively small (max(|**a_o_**|) = 0.0484, max(**K**) − 1 = 0.0263). The *x*-axis has the largest misalignment angle (max(diag(**Ψ**)) = 1.3702°).

#### 5.2.3. Validation

**Figure 6.**
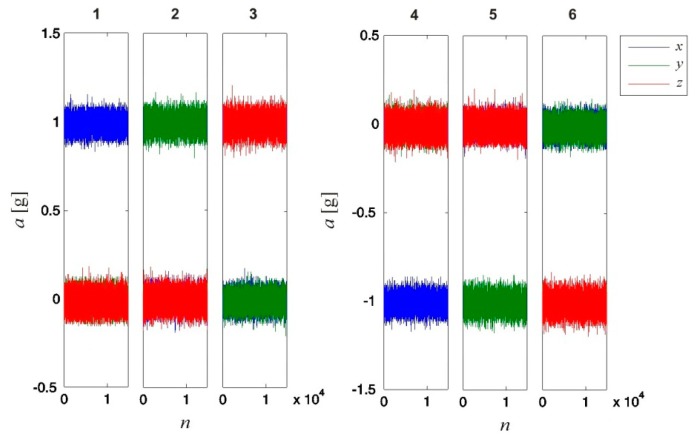
Calibration validation results. Six measurements were performed, and for each measurement, the values detected by the 3D accelerometer were corrected using the obtained 3D accelerometer calibration parameters. The sensor orientations during the validation measurements were chosen to be the same as for the six calibration measurements; the sensor's intrinsic coordinate axes (*x*, *y* and *z*) were aligned in turn along (measurements 1, 2, and 3) and opposite to (measurements 4, 5, and 6) the direction of gravitational acceleration **g**. The data were captured at a sampling rate *f_s_* = 1000 Hz.

The calibration results were validated using another set of measurements. During these measurements, the sensor orientation was equal to that during the calibration measurements, and an equal number of samples were considered. The results are shown in Figure 6. By comparing the values presented in Figures 5 and 6, we can see that on average, the calibrated values correspond more accurately to the measured values (projections of 1 g on the sensor's intrinsic coordinate system axes). Due to the noisy sensor, the same effect of noise is observable in both figures.

### 5.3. 3D Gyroscope Calibration

The 3D gyroscope was calibrated in accordance with the procedure presented in Section 4.

#### 5.3.1. Measurements

**Figure 7.**
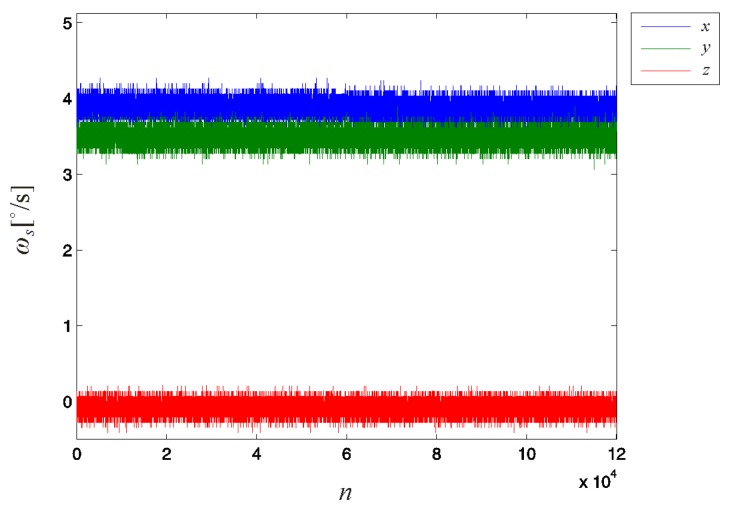
Values detected by the 3D gyroscope for the first calibration measurement, during which the sensor was held still in an arbitrary position. The data were captured at a sampling rate *f_s_* = 1000 Hz. The measurement is used to estimate the zero level offsets of the sensor sensitivity axes.

The calibration measurement required to estimate the zero level offset **ω_o_** of the 3D gyroscope (during which the sensor was held still) was performed over a duration of 120 s, yielding *N_off_* = 120,000 samples (at a sampling rate of *f_s_* = 1000 Hz), as shown in Figure 7.

For the remaining three measurements required to estimate the values of the calibration matrix **C_s_**, we attempted to apply an approximately average angular velocity of 100 °/s for each of the three manual rotations of the sensor around coordinate axes *x*, *y* and *z*. For each of the three measurements, we rotated the sensor by exactly *φ_k_* = 1080°. The numbers of collected samples were *N_x_* = 8801, *N_y_* = 9502 and *N_z_* = 11,600. The total measurement time for all four calibration measurements was approximately 2.5 min.

The calibration time was determined by considering the effect of the noise on the error in the estimated calibration parameters. The analytic solution presented in Appendix B shows that for the considered 3D gyroscope (±2000°/s range) and a calibration angular velocity 100°/s, the ratio of the power of the measurement noise to the variance of the error from the noisy calibration *NER* is larger than 6 dB for any value *N_off_* > 100,000 and *N_x_* = *N_y_* = *N_z_* > 8000.

**Figure 8.**
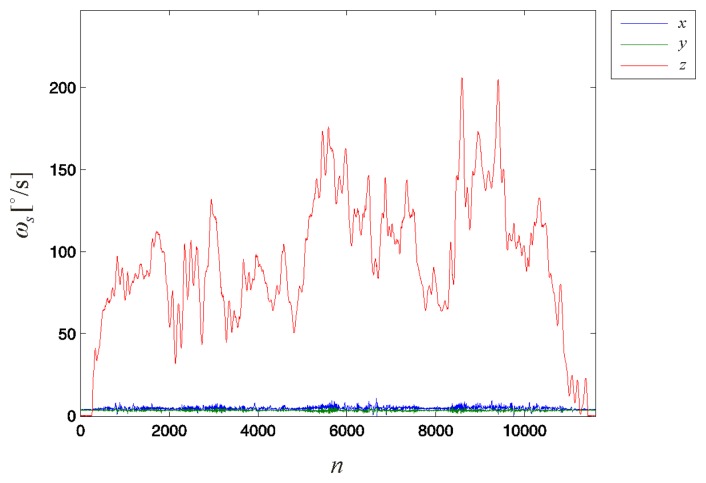
Values detected by the 3D gyroscope during sensor rotation around coordinate axis *z* for exactly three complete revolutions, *φ_k_* = 1080°. The data were captured at a sampling rate *f_s_* = 1000 Hz. Similar data were collected for the remaining two calibration measurements as the sensor was rotated around the *x*- and *y*-axes.

The number of samples required to achieve *NER* > 6dB is larger than that required for the 3D accelerometer investigated. Thus, we conclude that the 3D gyroscope calibration is more sensitive to noise than the 3D accelerometer. A larger number of samples is required for the 3D gyroscope than for the 3D accelerometer; thus, the 3D gyroscope calibration also requires a longer time than the 3D accelerometer, given the same *f_s_* for both sensors. The data collected during the rotation of the sensor around coordinate axis *z* are shown in Figure 8 as an example.

The observable variation in angular velocity is a consequence of the manual rotation of the 3D gyroscope. As long as the rotation axis does not change, this variation does not influence the calibration. A constant rotation axis was achieved using the specially designed casing for the device.

The 3D gyroscope investigated produces 16-bit outputs within the ±2000 °/s range. Each LSB thus accounts for 0.061 °/s. The variance of the quantization noise is (0.061°/s)^2^/12 = 0.00031 (°/s)^2^. The estimated variance of the sensor noise from the measurements is 0.010 (°/s)^2^. The total variation of the noise is equal to the sum of both variances, showing that that the variance of the quantization noise is negligible in this case.

For *N* measurement samples, the variance of the noise is reduced by the factor *N*. *N*=8801 samples were averaged over because this value corresponded to the lowest number of samples that was collected among all of the four calibration measurements. This result was used to calculate the highest total standard deviation of the noise of the 3D gyroscope investigated of $\sqrt{0.01/8801}{}^{\circ}/s=0.0011{}^{\circ}/s$. We can conclude that the 3D gyroscope measurements are accurate up to two decimal places. These values are meaningful up to three decimal places. We present the 3D gyroscope calibration results at this precision.

#### 5.3.2. Results

We obtained the following vector of the zero level offsets for the sensitivity axes by averaging the samples obtained when the sensor was at rest (the first calibration measurement):

(36) $${\text{ω}}_{\text{o}}=\left[\begin{array}{c} 3.871\\ 3.483\\ -0.085 \end{array}\right]$$

Averaging the samples captured during the sensor rotations around the three coordinate system axes (the remaining three calibration measurements) yielded the following matrix of the average detected angular velocities:

(37) $${\text{Ω}}_{\text{s}}=\left[\begin{array}{ccc} 125.495 & 5.191 & 4.517\\ 2.112 & 115.026 & 3.212\\ -0.802 & -0.527 & 92.608 \end{array}\right]$$

The matrix of the average actual angular velocities while rotating the sensor around the three coordinate system axes obtained by considering the number of collected samples *N_x_*, *N_y_*, and *N_z_*, the known total rotation angle *φ_k_*, and the sample frequency *f_s_* is equal to:

(38) $$\text{Ω}=\left[\begin{array}{ccc} \frac{\varphi_{k}}{N_{x}}\cdot f_{vz0} & 0 & 0\\ 0 & \frac{\varphi_{k}}{N_{y}}\cdot f_{vz0} & 0\\ 0 & 0 & \frac{\varphi_{k}}{N_{x}}\cdot f_{vz0} \end{array}\right]=\left[\begin{array}{ccc} 122.713 & 0 & 0\\ 0 & 113.660 & 0\\ 0 & 0 & 93.103 \end{array}\right]$$

Inserting values of **ω_o_** Equation (36), **Ω_s_** Equation (37), and **Ω** Equation (38) into Equation (27) yields the following results for the calibration matrix and zero level offset vector:

(39) $${\text{C}}_{\text{s}}=\left[\begin{array}{ccc} 1.009 & -0.012 & 0.007\\ 0.012 & 1.019 & 0.003\\ 0.006 & 0.004 & 1.005 \end{array}\right]$$

The matrices of the 3D gyroscope sensitivities **K** and orientation **V** obtained using the values of **C_s_** Equation (39) and considering the norms of the directional vectors Equations (9)–(11) are:

(40) $$\text{K}=\left[\begin{array}{ccc} 0.991 & 0 & 0\\ 0 & 0.981 & 0\\ 0 & 0 & 0.996 \end{array}\right]$$

(41) $$\text{V}=\left[\begin{array}{ccc} 1.000 & 0.012 & 0.007\\ -0.01 & 1.000 & 0.003\\ -0.006 & -0.004 & 1.000 \end{array}\right]$$

The matrix of angles between the sensor coordinate and sensitivity axes **Ψ** that follows from **V** Equation (41) is:

(42) $$\text{Ψ}=\text{acos}(\text{V})=\left[\begin{array}{ccc} 0.782 & 89.329 & 89.600\\ 90.652 & 0.674 & 89.830\\ 90.336 & 90.224 & 0.404 \end{array}\right]$$

In the above expression, all angles are given in degrees. The rows of matrix **Ψ** represent the sensor sensitivity axes, and its columns represent the sensor coordinate axes.

Equations (36), (40), and (42) demonstrate that for axes *x* and *y*, the zero level offset is relatively large (|ω*_ox_*| = 3.871≫|ω*_oz_*| = 0.085, |ω*_oy_*| = 3.483≫|ω*_oz_*| = 0.085), and the inaccuracies in the sensitivities and orientations of the axes are comparable.

A comparison of both tested sensors indicates that the axes of the 3D gyroscope are less misaligned and exhibit more accurate sensitivities. The maximum relative zero level offset (the maximum zero level offset divided by the set measurement range of the sensor) is comparable for both devices ( $\frac{\left|\omega_{ox}\right|}{2000}=\frac{3.871}{2000}=0.002\approx \frac{\left|a_{oz}\right|}{24}=\frac{0.0484}{24}=0.0020$).

#### 5.3.3. Validation

**Figure 9.**
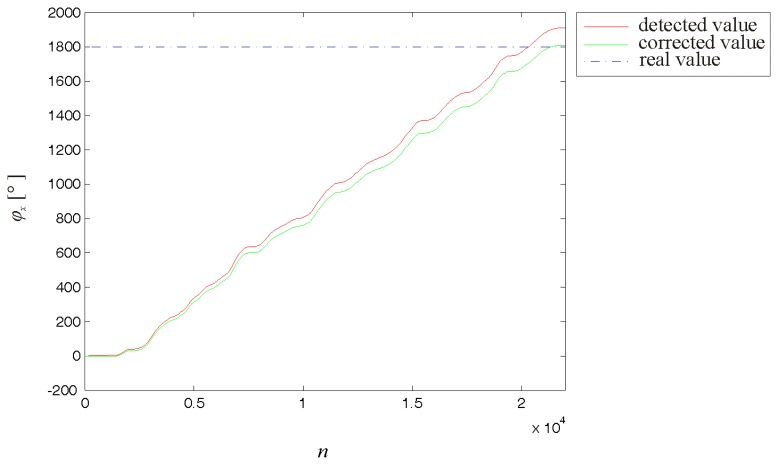
Calibration validation results. During the validation measurement, the sensor was rotated around its intrinsic axis *x* for five complete revolutions, *φ_x_* = 1800°. The data were captured at a sampling rate *f_s_* = 1000 Hz. The accuracy of the calculated rotation angle can be improved by correcting the detected values using the obtained calibration parameters.

To validate the calibration results, another measurement was performed in which the sensor was rotated on an even horizontal surface for five complete revolutions around its intrinsic axis *x* (*φ_x_* = 1800°). The values detected by the sensor were corrected using the established calibration parameters. Figure 9 presents the improvement in the accuracy of the rotation angle obtained by integrating the corrected values of the detected angular velocities.

## 6. Conclusions

The proposed 3D accelerometer and 3D gyroscope calibration procedures strive to compensate for inaccuracy in a simple and efficient manner (in terms of time and computational complexity) without affecting the benefits of using MEMS sensors, *i.e.*, simple, frequent, and low-cost everyday motion data capture.

The proposed calibration allows for the static compensation of the zero level offset, inaccurate sensitivity, and misalignment of the sensor sensitivity axes. The method operates on a small number of measurements, 6 for the 3D accelerometer and 4 for the 3D gyroscope. All calibration measurements are performed by manually positioning and rotating the sensor, and hence, no additional equipment is required for this task. The values of the 12 required calibration parameters are established using these measurements, and these parameters are used in further measurements to correct the value detected by the sensor. Correcting each detected 3D value requires nine addition operations and nine multiplication operations. Thus, data capture with an example capture frequency of 1000 samples per second requires 9000 addition operations and 9000 multiplication operations per second.

The calibration procedures presented in this paper are sufficiently simple that they can be easily performed each time a significant change in the sensor operating temperature occurs. Compensating for sensor inaccuracies at a specific operating temperature prevents temperature deviations of the outputs.

Both sensor calibrations rely on a generalized noiseless model of the 3D sensor that enables proper interpretation of the values detected by each sensor. According to this model, the values detected by the sensor are the projections of the measured value on the three sensitivity axes of the device. Although this finding is trivial for the 3D accelerometer, its validity had yet to be confirmed for the 3D gyroscope sensor example (for which this observation is not immediately apparent).

The effect of noise is neglected in the 3D sensor model presented here. However, the noise that occurs in practice introduces an error in the estimated values of the 12 calibration parameters. We can reduce the power of the noise by averaging over multiple samples that are collected for each calibration measurement. We can ensure that an adequate number of samples collected by requiring that the error from the calibration is lower than the error from the measurement noise.

The total calibration time depends on the number of calibration measurement samples and the sampling frequency. The number of required measurement samples for calibration is determined by considering the effects of noise. For the considered example sensor device (3D accelerometer range ±24 g and 3D gyroscope range ±2000°/s) the calibration parameters are established using calibration measurements of approximately 1.5 min in duration for the 3D accelerometer and 2.5 min in duration for the 3D gyroscope. The noise that affects the calibration over this period is substantially lower than the noise that affects each individual measurement for the sensors investigated. The 3D gyroscope requires a longer calibration time than the 3D accelerometer because the 3D gyroscope is more sensitive to noise than the 3D accelerometer.

The proposed procedure provides for a simple calibration process aimed at everyday usage and avoids the limitations of computationally complex and time-consuming calibration or the requirement of additional expensive equipment and training. Therefore, the proposed procedure enables the currently available low-cost MEMS sensors to be fully utilized.

## Appendix

### Appendix A. The Effect of Noise on the Calibration of a 3D Accelerometer

To evaluate the effect of noise on the calibration of a 3D accelerometer, we assume that the noise is the only source of error. We write:

(A1) $${\text{a}}_{\text{s}}=\text{a}+\text{ɛ}$$

where **a** is the vector of the measured acceleration in the sensor coordinate system and **ε** is the vector of the measurement noise that corresponds to a random stationary process.

The term **A_s_**_+_ denotes a 3 × 3 matrix of accelerations that is measured by the noisy sensor when its coordinate axes show in turn in the direction of the gravitational acceleration **g** (the first three calibration measurements). The term **A_s_**_−_ denotes a 3 × 3 matrix of measured acceleration values that are measured by the noisy sensor when its coordinate axes show in turn in the direction opposite to **g** (the remaining three calibration measurements). We write:

(A2) $$\begin{array}{ll} {\text{A}}_{\text{s}+} & =\left[\begin{array}{ccc} {\text{a}}_{\text{s},\;\text{x}+} & {\text{a}}_{\text{s},\;\text{y}+} & {\text{a}}_{\text{s},\;\text{z}+} \end{array}\right]\\ & =\left[\begin{array}{ccc} {\text{a}}_{\;\text{x}}+{\text{ɛ}}_{\text{x}+} & {\text{a}}_{\text{y}}+{\text{ɛ}}_{\text{y}+} & {\text{a}}_{\;\text{z}}+{\text{ɛ}}_{\text{z}+} \end{array}\right]\\ & =\text{A}+\left[\begin{array}{ccc} {\text{ɛ}}_{\text{x}+} & {\text{ɛ}}_{\text{y}+} & {\text{ɛ}}_{\text{z}+} \end{array}\right] \end{array}$$

(A3) $$\begin{array}{ll} {\text{A}}_{\text{s}-} & =\left[\begin{array}{ccc} {\text{a}}_{\text{s},\;\text{x}-} & {\text{a}}_{\text{s},\;\text{y}-} & {\text{a}}_{\text{s},\;\text{z}-} \end{array}\right]\\ & =\left[\begin{array}{ccc} -{\text{a}}_{\;\text{x}}+{\text{ɛ}}_{\text{x}-} & -{\text{a}}_{\text{y}}+{\text{ɛ}}_{\text{y}-} & -{\text{a}}_{\;\text{z}}+{\text{ɛ}}_{\text{z}-} \end{array}\right]\\ & =-\text{A}+\left[\begin{array}{ccc} {\text{ɛ}}_{\text{x}-} & {\text{ɛ}}_{\text{y}-} & {\text{ɛ}}_{\text{z}-} \end{array}\right] \end{array}$$

where **ε_x_**_+_, **ε_y_**_+_, **ε_z_**_+_, **ε_x_**_−_, **ε_y_**_−_ and **ε_z_**_−_ denote the vectors of the measurement noise for each of the six calibration measurements. The term **A** denotes a 3 × 3 matrix of the measured acceleration vectors for the first three calibration measurements. During each calibration measurement, an acceleration of 1 g is measured such that:

(A4) A=I

where **I** is the 3 × 3 identity matrix.

We merge the vectors of the measurement noise into two matrices, one for each measurement triplet:

(A5) $${\text{E}}_{\text{+}}=\left[\begin{array}{ccc} {\text{ɛ}}_{\text{x}+} & {\text{ɛ}}_{\text{y}+} & {\text{ɛ}}_{\text{z}+} \end{array}\right]$$

(A6) $${\text{E}}_{-}=\left[\begin{array}{ccc} {\text{ɛ}}_{\text{x}-} & {\text{ɛ}}_{\text{y}-} & {\text{ɛ}}_{\text{z}-} \end{array}\right]$$

Inserting the noisy measurements **A_s_**_+_ from Equation (A2) and **A_s_**_−_ from Equation (A3) into Equation (18) yields:

(A7) $$\begin{array}{cc} {\text{C}}_{\text{s}} & =2{\left({\text{A}}_{\text{s}+}-{\text{A}}_{\text{s}-}\right)}^{-1}\\ & =2\;{\left(2\;\text{I}+{\text{E}}_{\text{+}}-{\text{E}}_{-}\right)}^{-1} \end{array}$$

Inserting the noisy measurements **A_s_**_+_ from Equation (A2) and **A_s_**_−_ from Equation (A3) into Equation (20) yields:

(A8) $$\begin{array}{cc} {\text{a}}_{\text{o}} & =\frac{{\text{A}}_{\text{s}+}+{\text{A}}_{\text{s}-}}{6}\cdot \text{i}\\ & =\frac{{\text{E}}_{+}+{\text{E}}_{-}}{6}\cdot \text{i} \end{array}$$

To estimate the effect of the noise on the calibration, we assume that the only source of error is the error from the estimated calibration parameters (**C_s_** and **a_o_**). Thus, we neglect the effect of the noise on the measurement and assume that the detected value is equal to the measured acceleration, *i.e.*, **a_s_** = **a**. The estimated measured acceleration **a**′ is then equal to:

(A9) a'=Cs⋅(a−ao)=2(2I+E+−E−)−1⋅(a−E++E−6⋅i)

To obtain an analytic solution to Equation (A9), we assume that the amplitude of the noise (*i.e.*, the elements of the vectors **ε_x_**_+_, **ε_y_**_+_, **ε_z_**_+_, **ε_x_**_−_, **ε_y_**_−_ and **ε_z_**_−_) is significantly smaller than the amplitude of the measured values. Thus, we can neglect all of the elements containing the product of two or more elements of the matrices **E**_+_ and **E**_−_ in the derivation. We used Wolfram Mathematica 7.0 [47] to obtain the following analytic solution to Equation (A9):

(A10) a'≈(I−12(E+−E−))⋅(a−E++E−6⋅i)=a−(12(E+−E−)⋅a−E++E−6⋅i)

We introduce the term var(ε) to denote the variance of the 3D accelerometer measurement noise and the term var(**a**) to denote the variance of the detected acceleration, *i.e.*, the variance that is obtained by correcting the measured acceleration using the calibration parameters that are estimated from a larger number of noisy calibrations. The term var(**a**) represents a vector. The elements of var(**a**) are equal to the variances of the respective elements of **a**. We assume that var(ε) is equal for all three sensor coordinate axes, such that all of the elements of **E**_+_ and **E**_−_ have the same variance. Thus, we conclude that the variances of all three components of **a**′ in Equation (A10) are the same.

We assume that the individual noises **ε_x_**_+_, **ε_y_**_+_, **ε_z_**_+_, **ε_x_**_−_, **ε_y_**_−_ and **ε_z_**_−_ are mutually independent of each other. If each individual noise is independent of the other noises, the variance of the noise is additive in the addition and subtraction of multiple noises. For simplicity, we only present the final result for var(**a**):

(A11) var(a)=var(a')≈3‖a‖2+16Navar(ɛ)⋅i

In the estimate of the variance of the measured acceleration given above, *N_a_* is the number of the measurement samples collected for each calibration measurement. The measured acceleration values are obtained by averaging over *N_a_* samples; thus, the variance of the noise is reduced by *N_a_*.

The results that are obtained from the simulations never exceed the values that are obtained analytically, considering Equation (A11).

We use Equation (A11) to obtain the following ratio between the power of the measurement noise and the variance of the error from the noisy calibration for each component of the measured value:

(A12) $$\begin{array}{cc} \text{NER} & =10\;\text{log}\left(\frac{\text{var}(ɛ)}{\text{var}(a)}\right)\\ & =10\text{log}(\frac{6N_{a}}{3{\left\|\text{a}\right\|}^{2}+1}) \end{array}$$

### Appendix B. The Effect of Noise on the Calibration of a 3D Gyroscope

To evaluate the effect of noise on the calibration of a 3D gyroscope, we assume that the noise is the only source of error. We write:

(B1) $${\text{ω}}_{\text{s}}=\text{ω}+\text{ɛ}$$

where **ω** is the vector of the measured angular velocity in the sensor coordinate system, and **ε** is the vector of the measurement noise that corresponds to a random stationary process.

In the noisy calibration measurements, the vector of the zero level offsets **ω_o_** that is obtained when the sensor is at rest (the firs calibration measurement) is:

(B2) $${\text{ω}}_{\text{o}}={\text{ɛ}}_{\text{o}}$$

The term **Ω_s_** denotes a 3 × 3 matrix of angular velocities that is measured with a noisy sensor during the three calibration measurements when the sensor is rotated around the *x*, *y* and *z* coordinate axes (the remaining three calibration measurements):

(B3) $${\text{Ω}}_{\text{s}}=\text{Ω}+\left[\begin{array}{ccc} {\text{ɛ}}_{\text{x}} & {\text{ɛ}}_{\text{y}} & {\text{ɛ}}_{\text{z}} \end{array}\right]$$

where **ε_x_**, **ε_y_** and **ε_z_** denote the noise vectors for each of the three calibration measurements, and **Ω** denotes a 3 × 3 matrix for the rotation angular velocities for these three calibration measurements:

(B4) $$\text{Ω}=\left[\begin{array}{ccc} \omega_{k,\;x} & 0 & 0\\ 0 & \omega_{k,\;y} & 0\\ 0 & 0 & \omega_{k,\;z} \end{array}\right]$$

For clarity, we introduce the following denotation:

(B5) $${\text{E}}_{\Omega }=\left[\begin{array}{ccc} {ɛ}_{\text{x}} & {ɛ}_{\text{y}} & {ɛ}_{\text{z}} \end{array}\right]-{ɛ}_{\text{o}}\cdot {\text{i}}^{\text{T}}$$

We assume that the amplitude of the noise (*i.e.*, the elements of the vectors **ε_0_**, **ε_x_**, **ε_y_** and **ε_z_**) is significantly smaller than the amplitude of the measured angular velocity:

(B6) $$\left|{ɛ}_{\Omega }\right|\ll \text{min}(\text{diag}(\text{Ω}))$$

Using the noisy measurements **ω_o_** in Equation (B2), **Ω_s_** in Equation (B3) and Equation (27), we obtain the following calibration matrix:

(B7) $$\begin{array}{cc} {\text{C}}_{\text{s}} & =\text{Ω}\cdot {\left({\text{Ω}}_{\text{s}}-{\text{ω}}_{\text{o}}\cdot {\text{i}}^{T}\right)}^{-1}\\ & =\text{Ω}\cdot {\left(\text{Ω}+{\text{E}}_{\text{Ω}}\right)}^{-1} \end{array}$$

To estimate the effect of the noise on the calibration, we assume that the only source of error is the error from the estimated calibration parameters (**C_s_** and **ω_o_**). Thus, we neglect the effect of the noise on the measurement and assume that the detected value is equal to the measured angular velocity, *i.e.*, **ω_s_** = **ω**. The estimated measured angular velocity **ω**′ is then equal to:

(B8) ω'=Cs⋅(ω−ωo)=Ω⋅(Ω+EΩ)−1(ω−ɛo)

To obtain an analytic solution to Equation (B8), we assume that the amplitude of the noise is significantly smaller than the amplitude of the measured values. Thus, all of the elements containing the product of two or more elements of the matrix **E_Ω_** can be neglected.

We further assume that the individual noises **ε_0_**, **ε_x_**, **ε_y_** and **ε_z_** are mutually independent of each other. If each individual noise is independent on the other noises, the variance of the noise is additive in the addition and subtraction of multiple noises.

We obtained the analytical solution for **ω**′ in Equation (B8) in terms of the individual elements of the matrices **E_Ω_** and **Ω** using Wolfram Mathematica 7.0 [47]. For simplicity, we only present the final results for the variances of each measured angular velocity component:

(B9) $$\begin{array}{l} \text{var}(\omega_{x})=\text{var}(\omega {'}_{x})\approx \frac{\text{var}(ɛ)}{N_{\text{off}}}\left({\left(1-\frac{\omega_{y}}{\omega_{k,\;y}}-\frac{\omega_{z}}{\omega_{k,\;z}}\right)}^{2}+{\left(\frac{\omega_{x}}{\omega_{k,\;y}}\right)}^{2}+{\left(\frac{\omega_{x}}{\omega_{k,\;z}}\right)}^{2}\right)+\\ +\frac{\text{var}(ɛ)}{N_{y}}\left({\left(\frac{\omega_{x}}{\omega_{k,\;y}}\right)}^{2}+{\left(\frac{\omega_{y}}{\omega_{k,\;y}}\right)}^{2}\right)+\frac{\text{var}(ɛ)}{N_{z}}\left({\left(\frac{\omega_{x}}{\omega_{k,\;z}}\right)}^{2}+{\left(\frac{\omega_{z}}{\omega_{k,\;z}}\right)}^{2}\right) \end{array}$$

(B10) $$\begin{array}{l} \text{var}(\omega_{y})=\text{var}(\omega {'}_{y})\approx \frac{\text{var}(ɛ)}{N_{\text{off}}}\left({\left(1-\frac{\omega_{x}}{\omega_{k,\;x}}-\frac{\omega_{z}}{\omega_{k,\;z}}\right)}^{2}+{\left(\frac{\omega_{y}}{\omega_{k,\;x}}\right)}^{2}+{\left(\frac{\omega_{\;y}}{\omega_{k,\;z}}\right)}^{2}\right)+\\ +\frac{\text{var}(ɛ)}{N_{x}}\left({\left(\frac{\omega_{x}}{\omega_{k,\;x}}\right)}^{2}+{\left(\frac{\omega_{y}}{\omega_{k,\;x}}\right)}^{2}\right)+\frac{\text{var}(ɛ)}{N_{z}}\left({\left(\frac{\omega_{y}}{\omega_{k,\;z}}\right)}^{2}+{\left(\frac{\omega_{\;z}}{\omega_{k,\;z}}\right)}^{2}\right) \end{array}$$

(B11) $$\begin{array}{l} \text{var}(\omega_{z})=\text{var}(\omega {'}_{z})\approx \frac{\text{var}(ɛ)}{N_{\text{off}}}\left({\left(1-\frac{\omega_{x}}{\omega_{k,\;x}}-\frac{\omega_{y}}{\omega_{k,\;y}}\right)}^{2}+{\left(\frac{\omega_{z}}{\omega_{k,\;x}}\right)}^{2}+{\left(\frac{\omega_{z}}{\omega_{k,\;y}}\right)}^{2}\right)+\\ +\frac{\text{var}(ɛ)}{N_{x}}\left({\left(\frac{\omega_{x}}{\omega_{k,\;x}}\right)}^{2}+{\left(\frac{\omega_{z}}{\omega_{k,\;x}}\right)}^{2}\right)+\frac{\text{var}(ɛ)}{N_{y}}\left({\left(\frac{\omega_{\;y}}{\omega_{k,\;y}}\right)}^{2}+{\left(\frac{\omega_{z}}{\omega_{k,\;y}}\right)}^{2}\right) \end{array}$$

In the estimates given above, *N_off_* is the number of samples collected during the first calibration measurement when the sensor is at rest. *N_x_*, *N_y_* and *N_z_* are the numbers of samples collected during the remaining three calibration rotations when the sensor is rotated around the *x*, *y* and *z* coordinate axes, respectively. The measured angular velocity values are obtained by averaging over *N_off_*, *N_x_*, *N_y_* and *N_z_* samples; thus, the variance of the noise is reduced by the number of samples.

The results that are obtained from the simulations never exceed the values that are obtained analytically, considering Equations (B9)–(B11).

If the number of samples collected, the calibration angular velocities and the measurement angular velocities are equal for each of the three calibration rotations, *i.e.*, *N_x_* = *N_y_* = *N_z_* = *N_r_*, ω*_x_* = ω*_y_* = ω*_z_* = ω, and ω*_k,x_* = ω*_k,y_* = ω*_k,z_* = ω*_k_* the variances for all the three components Equations (B9)–(B11) are equal. In this case, we obtain the following ratio for the power of the measurement noise to the variance of the error from the noisy calibration:

(B12) $$\begin{array}{cc} \text{NER} & =10\;\text{log}\left(\frac{\text{var}(ɛ)}{\text{var}(\omega )}\right)\\ & =10\text{log}(\frac{N_{\text{off}}\;N_{r}\;\omega_{k}^{2}}{4N_{\text{off}}\omega^{2}+N_{r}\;(\omega_{k}^{2}-4\;\omega \;\omega_{k}+6\;\omega^{2})}) \end{array}$$

## References

[1] Fleming W. (2008). New automotive sensors—A review. IEEE Sens. J..

[2] Weinberg H. (2002). MEMS sensors are driving the automotive industry. Sensors.

[3] Sparks D.R., Zarabadi S.R., Johnson J.D., Jiang Q., Chia M., Larsen O., Higdon W., Castillo-Borelley P. A CMOS Integrated Surface Micromachined Angular Rate Sensor: Its Automotive Applications.

[4] Tsai C.W., Chen K.H., Shen C.K., Tsai J.C. (2012). A MEMS Doubly Decoupled Gyroscope with Wide Driving Frequency Range. IEEE Trans. Ind. Electron..

[5] Trifunovic M., Vadiraj A.M., van Driel W.D. MEMS accelerometers and their bio-applications.

[6] Lin P.C., Komsuoglu H., Koditschek D.E. (2006). Sensor data fusion for body state estimation in a hexapod robot with dynamical gaits. IEEE Trans. Robot..

[7] McIlwraith D., Pansiot J., Yang G.Z. Wearable and ambient sensor fusion for the characterisation of human motion.

[8] Liebermann D.G., Berman S., Weiss P.L., Levin M.F. (2012). Kinematics of Reaching Movements in a 2-D Virtual Environment in Adults With and Without Stroke. IEEE Trans. Neural Syst. Rehabil. Eng..

[9] Aminian K., Robert P., Buchser E.E., Rutschmann B., Hayoz D., Depairon M. (1999). Physical activity monitoring based on accelerometry: Validation and comparison with video observation. Med. Biol. Eng. Comput..

[10] Aminian K., Najafi B. (2004). Capturing human motion using body-fixed sensors: Outdoor measurement and clinical application. Comput. Animat. Virtual Worlds.

[11] Najafi B., Aminian K., Loew F., Blanc Y., Robert P.A. (2002). Measurement of stand-sit and sit-stand transitions using a miniature gyroscope and its application in fall risk evaluation in the elderly. IEEE Trans. Biomed. Eng..

[12] Uiterwaal M., Glerum E.B.C., Busser H.J., Lummel R.C. (1998). Ambulatory monitoring of physical activity in working situations, a validation study. J. Med. Eng. Technol..

[13] Ayrulu-Erdem B., Barshan B. (2011). Leg motion classification with artificial neural networks using wavelet-based features of gyroscope signals. Sensors.

[14] Park S.K., Suh Y.S. (2010). A zero velocity Detection Algorithm Using Inertial Sensors for Pedestrian Navigation Systems. Sensors.

[15] Tunçel O., Altun K., Barshan B. (2009). Classifying human leg motions with uniaxial piezoelectric gyroscopes. Sensors.

[16] Roetenberg D., Slycke P.J., Veltink P.H. (2007). Ambulatory Position and Orientation Tracking Fusing Magnetic and Inertial Sensing. IEEE Trans. Biomed. Eng..

[17] Stančin S., Tomažič S. (2013). Early Improper Motion Detection in Golf Swings Using Wearable Motion Sensors: The First Approach. Sensors.

[18] Hoffman M., Varcholik P., LaViola J.J. Breaking the status quo: Improving 3D Gesture Recognition with Spatially Convenient Input Devices.

[19] Schall G., Wagner D., Reitmayr G., Taichmann E., Wieser M., Schmalstieg D., Hofmann-Wellenhof B. Global Pose Estimation Using Multi-Sensor Fusion for Outdoor Augmented Reality.

[20] Su C., Xu W., Mengnan G., Shengquan Y. Simulation Teaching in 3D Augmented Reality Environment.

[21] Kawasaki H., Nakayama K., Parker G. Teaching for multi-fingered robots based on motion intention in virtual reality.

[22] Tomažič S., Stančin S. (2011). Simultaneous orthogonal rotation angle. Electrotech. Rev..

[23] Stančin S., Tomažič S. (2011). Angle Estimation of Simultaneous Orthogonal Rotations from 3D Gyroscope Measurements. Sensors.

[24] Giansanti D., Maccioni G. Guidelines for Calibration and Drift Compensation of a Wearable Device with Rate-Gyroscopes and Accelerometers.

[25] Feng S., Fengli L., Xirui F. A new method of zero calibration of the strapdown inertial navigation system.

[26] Won S., Golnaraghi F. (2010). A triaxial accelerometer calibration method using a mathematical model. IEEE Trans. Instrum. Meas..

[27] Camps F., Harasse S., Monin A. Numerical calibration for three axis accelerometers and magnetometers.

[28] Wang J., Liu Y., Fan W. Design and calibration of a smart inertial measurement unit for autonomous helicopters using MEMS sensors.

[29] Kim A., Golnaraghi M. Initial calibration of an inertial measurement unit using an optical position tracking system.

[30] Jurman D., Jankovec M., Kamnik R., Topič M. (2007). Calibration and data fusion solution for the miniature attitude and heading reference system. Sens. Actuators A Phys..

[31] Renk E.L., Rizzo M., Collins W., Lee F., Bernstein D.S. (2005). Calibrating a triaxial accelerometer-magnetometer—Using robotic actuation for sensor reorientation during data collection. IEEE Trans. Control Syst. Technol..

[32] Vcelak J., Ripka P., Kubik J., Platil A., Kaspar P. (2005). AMR navigation systems and methods of their calibration. Sens. Actuators A Phys..

[33] Petrucha V., Kaspar P. Calibration of a triaxial fluxgate magnetometer and accelerometer with an automated non-magnetic calibration system.

[34] Zhu R., Zhou Z. (2006). Calibration of three-dimensional integrated sensors for improved system accuracy. Sens. Actuators A Phys..

[35] Beravs T., Podobnik J., Munih M. (2012). Three-Axial Accelerometer Calibration Using Kalman Filter Covariance Matrix for Online Estimation of Optimal Sensor Orientation. IEEE Trans. Intsrum. Meas..

[36] Li W., Du Q., Mi P. A MEMS inertial sensor and AMR magnetic sensor calibration method.

[37] Fei J., Zhou J. (2012). Robust Adaptive Control of MEMS Triaxial Gyroscope Using Fuzzy Compensator. IEEE Trans. Syst. Man Cybern. Part B: Cybern..

[38] Leland R.P. (2006). Adapptive Control of a MEMS Gyroscope Using Lyapunov Mehods. IEEE Trans. Control Syst. Technol..

[39] Barshan B., Durrant-Whyte H.F. (1995). Inertial Navigation Systems for Mobile Robots. IEEE Trans. Robot. Autom..

[40] Wang J.H., Gao Y. (2010). Land vehicle dynamics-aided inertial navigation. IEEE Trans. Aerosp. Electron. Syst..

[41] Koifman M., Bar-Itzhack I.Y. (1999). Inertial navigation system aided by aircraft dynamics. IEEE Trans. Control Syst. Technol..

[42] Zhao L., Yan L., Cheng J., Wang X. The Research of Inertial Navigation System Based on Submarine Space Motion.

[43] Grenon G., An P.E., Smith S.M., Healey A.J. (2001). Enhancement of the inertial navigation system for the Morpheus autonomous underwater vehicles. IEEE J. Ocean. Eng..

[44] Woodman O.J. An Introduction to Inertial Navigation. http://www.cl.cam.ac.uk/techreports/UCAM-CL-TR-696.html.

[45] STMicroelectronics LIS331HH MEMS Digital Output Motion Sensor Ultra Low-Power High Full-Scale 3-Axes “nano” Accelerometer. http://www.st.com/web/catalog/sense_power/FM89/SC444/PF247976.

[46] InvenSense ITG3200–3 Integrated Triple-Axis Digital-Output Gyroscope. http://www.invensense.com/mems/gyro/itg3200.html.

[47] (2008). Mathematica.

